# Hereditary cancer screening: Case reports and review of literature on ten Ashkenazi Jewish founder mutations

**DOI:** 10.1002/mgg3.460

**Published:** 2018-08-27

**Authors:** Devin M. Cox, Katherine L. Nelson, Meera Clytone, Debra L. Collins

**Affiliations:** ^1^ University of Kansas Cancer Center Westwood Kansas

**Keywords:** *APC*, Ashkenazi, *BRCA1*, *BRCA2*, breast cancer, cascade testing, *CHEK2*, colon cancer, founder mutations, *GREM1*, Jewish, *MSH2*, *MSH6*

## Abstract

**Background:**

Historically, three founder mutations in the *BRCA1/2* (OMIM 113705; OMIM 600185) genes have been the focus of cancer risks within the Ashkenazi Jewish (AJ) population. However, there are several additional mutations associated with increased susceptibility to cancer in individuals of AJ ancestry.

**Methods:**

We report three patients who exemplify the need to keep these additional founder mutations in mind when pursuing hereditary cancer genetic testing of individuals in this population. All gene sequences in this paper were aligned to reference sequences based on human genome build GRCh37/UCSC hg19.

**Results:**

review of the literature discusses that the combined risk is 12.36%–20.83% forhaving 1 of the 10 hereditary cancer AJ founder mutations in the *BRCA1, BRCA2, CHEK2* (OMIM 604373)*, APC* (OMIM 611731)*, MSH2* (OMIM 609309)*, MSH6* (OMIM 600678)*,* and *GREM1* (OMIM 603054) genes for individuals of AJ ancestry.

**Conclusion:**

We recommend testing for all 10 of these AJ founder cancer susceptibility mutations for individuals within this population as standard screening in order to ensure appropriate cancer risk management and cascade testing.

## 1 INTRODUCTION

Individuals of Ashkenazi Jewish (AJ) ancestry are at increased risks for carrying certain genetic mutations due to the founder effect. Founder mutations can occur in isolated populations with limited influx of new genetic variants, leading to an increased prevalence of rare mutations (Ferla et al., 2007). The AJ population has multiple known founder mutations associated with hereditary conditions in the pediatric population which are typically evaluated through prenatal, pediatric, and adult genetics clinics. However, this paper focuses on genes associated with hereditary cancer risks (*BRCA1, BRCA2, CHEK2, APC, MSH2, MSH6,* and *GREM1*) that do not have pathognomonic features (i.e., the *BLM* gene which is associated with growth deficiency and a butterfly rash). Recognizing that an individual coming to a hereditary cancer clinic is of AJ descent can lead genetic counselors and other healthcare providers to offer genetic testing for all of the relevant AJ hereditary cancer founder mutations.

## 2 BREAST CANCER AND AJ ANCESTRY

Historically, individuals with AJ ancestry were offered *BRCA1/2* genetic analysis for three founder mutations, and if one of these was found, this was followed by site specific testing of other family members. These AJ founder mutations include two pathogenic mutations of the *BRCA1* gene (c.185delAG and c.5382insC) and one pathogenic mutation within the *BRCA2* gene (c.6174delT), these three mutations are found in approximately 1 in 40 individuals of AJ ancestry (Bahar et al., 2001; Ferla et al., 2007; Frank et al., 2002; Rosenthal, Moyes, Arnell, Evans, & Wenstrup, 2015).

One in 40 AJ individuals will have one of the three *BRCA1/2* founder mutations. These three mutations have an approximate 2%–3% frequency in AJ individuals : the *BRCA1* 185delAG mutation has a reported frequency of 0.96%–1.14%, the *BRCA1* 5382insC mutation has a 0.13%–0.28% frequency and BRCA2 6174delT has a 0.6%–1.52% frequency (Bahar et al., 2001; Ferla et al., 2007). Given the high frequency of these three founder mutations in the AJ population, testing for all three mutations is always recommended, even when a family has another known pathogenic hereditary cancer mutation.

With the advent of next‐generation panels which can perform analysis of multiple genes at once, sequencing and deletion duplication analysis of all variants of the *BRCA1/2* genes are now often offered to those with AJ ancestry. This testing can also include full analysis of other genes associated with hereditary breast cancer as well as genes with increased susceptibility to hereditary colon and other cancers.

There are also two AJ founder mutations in the *CHEK2* gene. Routine analysis of the *CHEK2* gene was initially limited to the c.1100delC mutation in this gene. Current screening guidelines for those who are gene positive refer primarily to this specific mutation which has a 0.06% frequency in the Caucasian population (Leedom et al., 2016). The frequency of this mutation has been seen equally in AJ and non‐AJ individuals (Offit et al., 2003; Shaag et al., 2005); and thus does not appear to have a founder effect in the AJ population.

However another *CHEK2* mutation, the c.1283C>T (p.Ser428Phe) mutation does have an increased frequency in the AJ population of between 2.4% and 5% (Laitman, Kaufman, Lahad, Papa, & Friedman, 2007; Leedom et al., 2016; Shaag et al., 2005; Walsh et al., 2017). Shaag et al. (2005) concluded that the S428F mutation conferred a 2‐fold increased breast cancer risk among AJ women. A second *CHEK2* mutation with an increased frequency within the AJ population is the c.470T>C (p.Ile157Thr) mutation which is found in 0.7% of the general Caucasian population, but it is reported in 0.46%–1.2% of the AJ population (Laitman et al., 2007; Leedom et al., 2016). Leedom et al. (2016) also concluded that the Ile157Thr mutation is a moderate risk mutation which confers a 1.5 fold increased risk for breast cancer compared to other *CHEK2* mutations. These two *CHEK2* founder mutations have an approximate 3%–4% frequency in the AJ population equating to approximately 1 in 30 AJ individuals having one of these two mutations. The National Comprehensive Cancer Network's (NCCN) guidelines regarding cancer risk and management for the *CHEK2* gene are based on frameshift mutations (Daly et al., 2018; Provenzale et al., 2017). The risks for missense mutations are unclear, but the breast cancer risk with the Ile157Thr mutation appears to be lower. Since the NCCN guidelines have not been established for all variants of *CHEK2* mutations, it may be prudent to consider screening recommendations for the known mutation, with consideration of personal and family history. Knowing which individuals inherited an AJ *CHEK2* founder mutation could help determine which family members could benefit from increased screening or surveillance. This knowledge will also become impactful in the future when further information regarding non frameshift pathogenic *CHEK2* mutation management guidelines become available.

**Figure 1:**
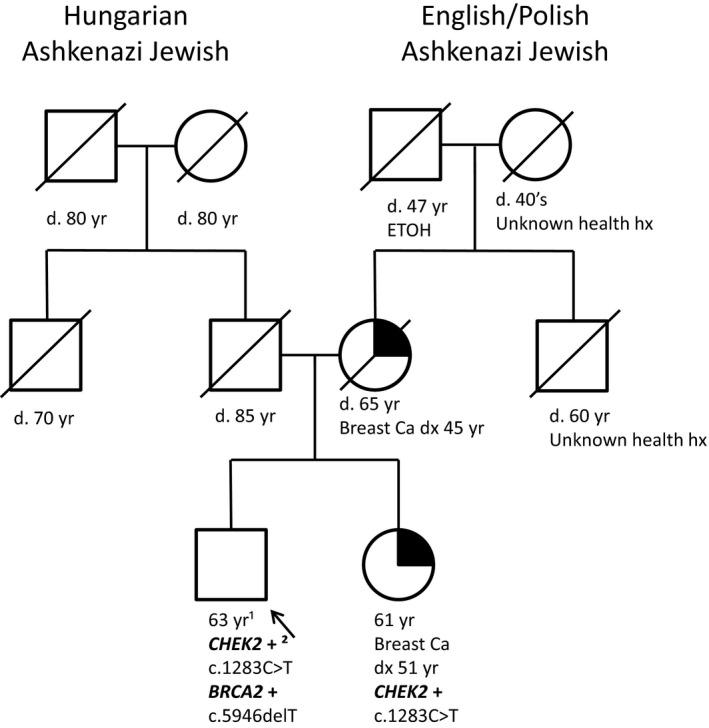
Pedigree of Patient 1. ¹Yr, ¹years; +², ²positive for pathogenic mutation

Our first patient was a 63 year old male with no personal history of cancer who presented to our clinic with a family history of a pathogenic *CHEK2* mutation (Figure 1). The patient's 61 year old sister was diagnosed with breast cancer at 51 years of age, she had previous multi‐gene panel testing performed, which showed the c.1283C>T pathogenic AJ founder mutation in the *CHEK2* gene. The relevant family history included a mother diagnosed with breast cancer at 45 years of age who had passed away and both maternal and paternal AJ lineages. We pursued *BRCA1, BRCA2* and *CHEK2* gene testing through Invitae Laboratory which found the c.1283C>T pathogenic *CHEK2* mutation as well as a c.5946delT pathogenic *BRCA2* mutation (aka 6174delT). If testing had pursued only the known familial pathogenic mutation this additional *BRCA2* founder mutation would have been missed.

**Figure 2:**
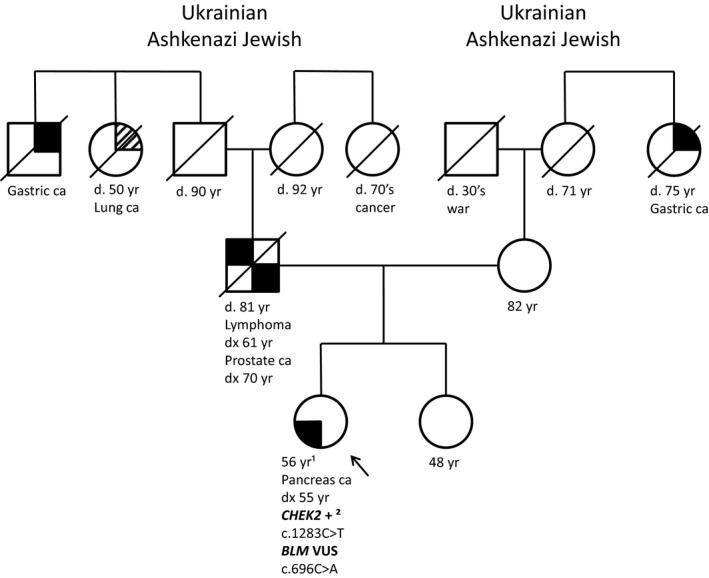
Pedigree of Patient 2. ¹Yr, ¹years; +², ²positive for pathogenic mutation

Our second patient was a 56 year old female who was diagnosed with pancreas cancer at 55 years of age who never smoked (Figure 2). The reported family history included a maternal great aunt with gastric cancer diagnosed at 75 years of age, a father diagnosed with lymphatic cancer at 61 years of age and prostate cancer at 70 years of age, and a paternal great uncle diagnosed with gastric cancer. Both maternal and paternal lineages included AJ ancestry. Several years prior to her diagnosis the patient was offered AJ founder mutation testing for the *BRCA1* and *BRCA2* genes but declined testing. Following her diagnosis of pancreatic cancer, she now elected to pursue genetic testing analysis including 79 genes on a multi cancer next generation panel through Invitae Laboratory. This test found the pathogenic AJ founder mutation in the *CHEK2* gene (c.1283C>T) as well as a variant of uncertain clinical significance in the *BLM* gene (c.696C>A). This patient illustrates the need for AJ population screening to include more than just the *BRCA1* and *BRCA2* AJ founder mutations. Had this patient only pursued the traditional *BRCA1/2* AJ founder mutations she may not have pursued additional genetic testing after being diagnosed with pancreas cancer and her *CHEK2* mutation could have been missed. This patient also demonstrates the importance of pursuing AJ population based screening as her family history is not indicative of the *CHEK2* mutation which was found.

It is important to not only offer the AJ population screening for the three known *BRCA1/2* AJ founder mutations, but also other pathogenic mutations associated with hereditary cancer within this population. Walsh et al. (2017) reported that AJ women diagnosed with invasive breast cancer, without a *BRCA1/2* founder mutation, have a remaining 1% chance of having a non‐founder *BRCA1/2* pathogenic mutation; if she is diagnosed before 40 years of age this chance increases to 3% (Walsh et al., 2017). Frank et al. (2002) found that 16 of the 74 (21.64%) deleterious *BRCA1/2* mutations found in AJ individuals were non‐founder mutations, supporting the need for full *BRCA1/2* gene sequencing in AJ individuals.

Clinical questions arise from these risk estimates. If an individual of AJ ancestry presents to a hereditary cancer clinic for population based genetic testing how many genes, or which founder mutations should be offered? Several papers provide data for consideration. Fifty percent of *BRCA1/2* positive women diagnosed with breast cancer had no known immediate family history of cancer, indicating family history alone may not be enough to determine appropriate testing (Shkedi‐Rafid, Gabai‐Kapara, Grinshpun‐Cohen, & Levy‐Lahad, 2012). Individuals from AJ families may present unique challenges in determining appropriate testing, in part due to smaller family sizes and lack of medical information concerning past generations due to factors such as the Holocaust (Shkedi‐Rafid et al., 2012). Specifically, no family historians may be living or available and family members may have died at young ages, prior to developing cancer. We therefore recommend for adding AJ founder mutations for genes associated with breast and colorectal cancer (*APC, MSH2, MSH6,* and *GREM1)* regardless of family history as standard screening within the AJ population.

## 3 COLORECTAL CANCER AND AJ ANCESTRY

Currently, the NCCN guidelines do not address testing AJ individuals for founder mutations in the colorectal cancer genes,even though, according to Raskin et al. (2011) approximately 14%–16% of all colorectal cancers within the AJ population can be attributed to founder mutations in the *APC, BLM, MSH2* and *MSH6* genes. The *GREM1* AJ founder mutation is a 40 kb duplication upstream of the gene and is present in 0.7% of AJ individuals meeting Lynch syndrome criteria (Laitman, Jaeger, Katz, Tomlinson, & Friedman, 2015). Laitman's (2015) single patient with this mutation did not fit the criteria for *GREM1* as they had no increased polyp production. The *MSH2* AJ founder mutation c.1906G>C (p.Ala636Pro) has a frequency of 0.4%–0.7% (Foulkes et al., 2002; Guillem et al., 2007; Lavie, Gruber, Lejbkowicz, Dishon, & Rennert, 2008). According to Foulkes et al. (2002) this mutation accounts for 2%–3% of all colorectal cancers diagnosed before 60 years of age within the AJ population, while Guillem et al. (2007) indicates that this mutation accounts for two thirds of the Lynch syndrome mutations within the AJ population. Foulkes et al. (2002) reported that 271 AJ women diagnosed with breast cancer and having a family history of either colorectal or ovarian cancer were tested for this founder mutation and no mutations were identified. The *MSH6* gene has two separate AJ founder mutations; the c.3984_3987dupGTCA has a 0.3% frequency in this population while the c.3959_3962delCAAG has a 0.11% frequency (Raskin et al., 2011). Raskin et al. (2011) found none of the 22 unrelated families with these two founder mutations met Amsterdam I criteria, and only two of these families met the Amsterdam II criteria.

The AJ *APC* founder mutation c.3920T>A (p.Ile1307Lys) has a frequency of 6.1%–12% (Bahar et al., 2001; Foulkes et al., 2002; Raskin et al., 2011). The *APC* AJ founder mutation is unique as it does not present with the typical presentation of familial adenomatous polyposis (FAP) syndrome or even of attenuated FAP syndrome, but rather appears to only increase the risk for colorectal cancers in the AJ population by creating a hyper mutable region of the gene (Bahar et al., 2001; Laken et al., 1997). The risk for individuals in the AJ population to have colorectal cancer with this *APC* AJ founder mutation is estimated to double the general population risk, but does not appear to increase the risk for non AJ individuals (Boursi et al., 2013; Liang et al., 2013). According to a study by Laken et al. (1997) the *APC* AJ founder mutation was found in 28% of AJ families with a family history of colorectal cancer.

The NCCN guidelines have screening and management recommendations for all of the AJ founder mutation in the colorectal cancer genes (*APC, GREM1, MSH2* and *MSH6*). Therefore, knowing an individual inherited one of these mutations would direct future medical management and surveillance. This would include beginning colonoscopy screenings earlier and repeating them more often than the average individual (Daly et al., 2018; Provenzale et al., 2017).

**Figure 3:**
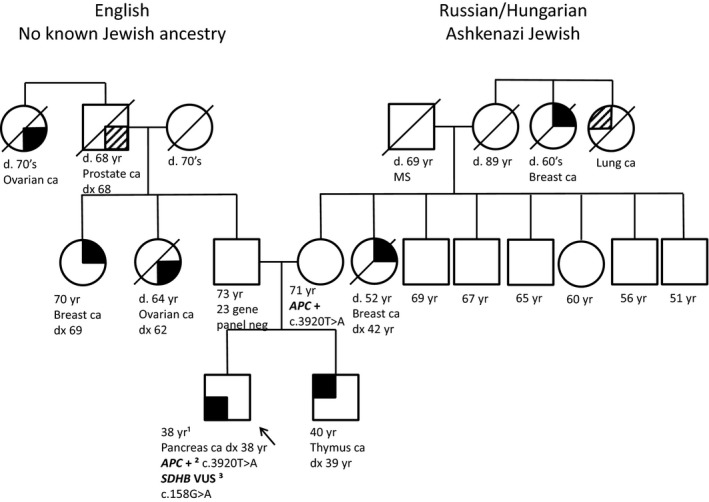
Pedigree of Patient 3. ¹Yr, ¹years; +², ²positive for pathogenic mutation; VUS³, ³variant of uncertain clinical significance

Our third patient had an incidental *APC* AJ founder mutation identified on genetic testing after he was diagnosed with pancreas cancer at 38 years of age (Figure 3). His family history included a brother with thymus cancer diagnosed at 39 years of age, a maternal aunt with breast cancer diagnosed at 42 years of age, a maternal great aunt with breast cancer who passed away in her 60s, a paternal aunt with ovarian cancer who passed away at 64 years of age, another paternal aunt with breast cancer diagnosed at 69 years of age; paternal grandfather had prostate cancer diagnosed at 68 years of age, a paternal great aunt had ovarian cancer and passed away in her 70's. The maternal ancestry was Russian and Hungarian with AJ ancestry. The paternal ancestry was United Kingdom with no known AJ ancestry. Results of the patient's 79 multi gene cancer panel through Invitae Laboratory found the AJ *APC* founder mutation (c.3920T>A) as well as a variant of uncertain clinical significance in the *SDHB* gene (c.158G>A). His mother (with AJ ancestry) subsequently had genetic testing from GeneDx Laboratory for the 20 gene breast ovarian cancer panel with the *APC* gene added on and was found to have the AJ *APC* founder mutation (c.3920T>A). Therefore, testing for the *APC* AJ founder mutation in AJ families is warranted, even without a family history of colorectal cancer.

## 4 DISCUSSION

AJ founder mutations for genes associated with hereditary cancer are prevalent in the AJ population with a frequency of approximately 12.36%–20.83% (Table 1). The incidence of *BRCA1*,* BRCA2* and *CHEK2* founder mutations are approximately 4.75%–7.02% and the *GREM1, MSH2, MSH6,* and *APC* founder mutations are approximately 7.61%–13.81%.

**Table 1:** Frequencies of AJ founder mutations in genes associated with cancer

Breast Cancer Genes		Colorectal Cancer Genes	
Mutation	Frequency	Mutation	Frequency
*BRCA1* 185delAG	0.96–1.14%	*GREM1* 40 kb dup	0.70%
*BRCA1* 5382insC	0.13–0.28%	*MSH2* 1906G>C	0.4–0.7%
*BRCA2* 6174delT	0.6–1.52%	*MSH6* 3984_3987dupGTCA	0.30%
*CHEK2* 1283C>T	2.6–2.88%	*MSH6* 3959_3962delCAAG	0.11%
*CHEK2* 470T>C	0.46–1.2%	*APC* 3920T>A	6.1–12%
Total	4.75–7.02%	Total	7.61–13.81%
Overall total mutation frequency 12.36%–20.83%^a^			

a For genes associated with increased risk for breast, ovary, uterus, colon, and other cancers.

These high gene frequencies, and our patient reports, illustrate the importance of incorporating evaluation and testing for common risk alleles within the AJ population as a part of hereditary cancer gene analysis as standard screening. These mutation are not only fairly frequent in this population, they can directly impact medical management and care for these families. Population based screening may provide early detection, screening, management and treatment for these associated hereditary cancer syndromes. Additional cascade testing of other family members can lead to effective risk‐reducing management and preventative decisions by providers. Seeing a genetic counselor or other provider with expertise in hereditary cancer syndromes is important for people with AJ ancestry. Those with expertise in hereditary cancer syndromes will be aware of these founder mutations and will discuss the option of pursuing a larger panel based genetic test which could include all of the AJ founder mutations. Genetic counselors and genetic specialists will also emphasize how screening and surveillance recommendations continue to evolve for these AJ founder mutations.

## CONFLICTS OF INTEREST

All authors have read and approved the submission to the journal. The authors report no conflicts of interest.
